# Diagnostic challenge of GAD65-associated autoimmune encephalitis mimicking post-traumatic complications in an older adult: a case report

**DOI:** 10.3389/fmed.2026.1929756

**Published:** 2026-08-26

**Authors:** Xiuqing Huang, Qile Zhang, Li Huang, Jian Xu

**Affiliations:** Department of Rehabilitation, Quzhou People’s Hospital; The Quzhou Affiliated Hospital, Wenzhou Medical University, Quzhou, Zhejiang, China

**Keywords:** autoimmune encephalitis, azathioprine, elderly patient, GAD65 antibody, intravenous immunoglobulin, traumatic brain injury

## Abstract

**Introduction:**

Glutamic acid decarboxylase 65 (GAD65) antibody-associated autoimmune encephalitis is an uncommon immune-mediated neurological disorder with heterogeneous neuropsychiatric manifestations. Diagnosis is particularly challenging in older adults when symptoms of recent traumatic brain injury (TBI) overlap with structural or metabolic conditions.

**Case presentation:**

We report a 79-year-old man who developed recurrent headache, progressive confusion, abnormal behavior, poor oral intake, intermittent myoclonic jerks, and low-grade fever after TBI complicated by chronic subdural hematoma. Initial laboratory testing revealed hyponatremia, and the working diagnosis was post-traumatic brain syndrome with syndrome of inappropriate antidiuretic hormone secretion (SIADH). Although serum sodium was corrected and nocturnal agitation partially improved, persistent daytime confusion, cognitive impairment, behavioral abnormalities, and low-grade fever prompted further investigation. Cerebrospinal fluid (CSF) analysis showed mild inflammatory changes with pleocytosis and elevated protein. CSF metagenomic next-generation sequencing (mNGS) and paraneoplastic antibody testing were negative, whereas the autoimmune encephalitis panel was positive for GAD65 antibody at a titer of 1:32. Whole-body positron emission tomography-computed tomography (PET-CT) did not reveal malignancy. The diagnosis was therefore revised to GAD65 antibody-associated autoimmune encephalitis. Because high-dose corticosteroids were considered unsuitable owing to his history of gastric ulcer and previous gastrointestinal bleeding, he was treated with intravenous immunoglobulin (IVIG) at a total dose of 2 g/kg divided over 3 consecutive days followed by oral azathioprine 50 mg daily. His fever resolved within 1 week, and his mental status and responsiveness gradually improved. At 6-month follow-up, he had achieved near-baseline mental status, was able to walk with a cane, and was almost fully independent in daily activities, without relapse or serious treatment-related adverse events.

**Discussion:**

This case highlights the diagnostic difficulty of recognizing autoimmune encephalitis after TBI. The clinical overlap between post- traumatic syndrome and immune-mediated brain injury often delays diagnosis, emphasizing the value of targeted CSF autoantibody testing in atypical cases.

**Conclusion:**

Traumatic brain injury may obscure the onset of GAD65-associated autoimmune encephalitis. Early recognition and a corticosteroid-sparing immunotherapy strategy (IVIG and azathioprine) can yield favorable outcomes in elderly patients with medical comorbidities.

## Introduction

Autoimmune encephalitis (AE) is an immune-mediated inflammatory disorder of the central nervous system characterized by autoantibodies against neuronal antigens (1). According to the location of the target antigen, AE can be broadly divided into neuronal surface antibody-associated and intracellular antigen-associated forms. The former includes N-methyl-D-aspartate receptor (NMDAR), leucine-rich glioma-inactivated 1 (LGI1), contactin-associated protein-like 2 (CASPR2), α-amino-3-hydroxy-5-methyl-4-isoxazolepropionic acid receptor (AMPAR), and γ-aminobutyric acid B receptor (GABABR) encephalitis, whereas the latter includes antibodies against intracellular antigens such as glutamic acid decarboxylase 65 (GAD65) and classical paraneoplastic antibodies including Hu, Yo, and Ri (1, 2). Compared with neuronal surface antibody-associated AE, intracellular antigen-associated AE often shows a more insidious onset, slower progression, and a poorer response to immunotherapy (2, 3). GAD65 antibody-associated AE is relatively uncommon, accounting for approximately 5%–10% of AE cases, and may occur across a wide age range. It is often associated with other autoimmune diseases and, less commonly, with underlying malignancy (3, 4). Clinical manifestations are heterogeneous and may include cognitive impairment, seizures, cerebellar ataxia, or stiff-person syndrome, and the overall prognosis is often unsatisfactory because response to conventional immunotherapy is limited (3, 5). Traumatic brain injury (TBI) has been proposed as a possible trigger of AE in some patients. A potential mechanism is disruption of the blood-brain barrier, which may expose previously sequestered neuronal antigens to the peripheral immune system and promote autoimmune responses (6, 7). However, a definite causal relationship has not been established, and the reported interval between TBI and AE onset usually ranges from weeks to months (6). Recent advances in the understanding of AE pathophysiology further support the possibility that such triggers may contribute to disease onset in selected cases (8). Here, we report an elderly man who developed progressive neuropsychiatric symptoms and cognitive decline during recovery from TBI and was subsequently diagnosed with GAD65 antibody-associated AE, highlighting the diagnostic challenge of identifying AE in the post-traumatic setting.

## Case presentation

A 79-year-old man weighing 48 kg was transferred to our hospital because of recurrent headache, progressive confusion, abnormal behavior, poor oral intake, and intermittent involuntary limb jerks after a recent traumatic brain injury. His history included coronary artery disease treated with coronary stent implantation 5 years earlier, although he had discontinued related medications 3 years before admission, and gastric ulcer complicated by gastrointestinal bleeding 7 years earlier. Before the traumatic event, he was independent in activities of daily living and had no known cognitive impairment, psychiatric disorder, seizures, diabetes mellitus, or other autoimmune disease. His family reported normal baseline neurological function. There was no known family history of autoimmune or neurological disease. Three months before admission, he fell while walking and developed headache. A local hospital diagnosed subdural hematoma and treated him conservatively, after which he substantially recovered. Ten days before admission, recurrent moderate headache, fatigue, and reduced energy developed, and he was rehospitalized locally for suspected chronic subdural hematoma. During hospitalization, he gradually developed nocturnal shouting, agitation, restlessness, poor oral intake, and intermittent involuntary limb jerks, and eventually became unable to get out of bed or walk independently.

**FIGURE 1:**
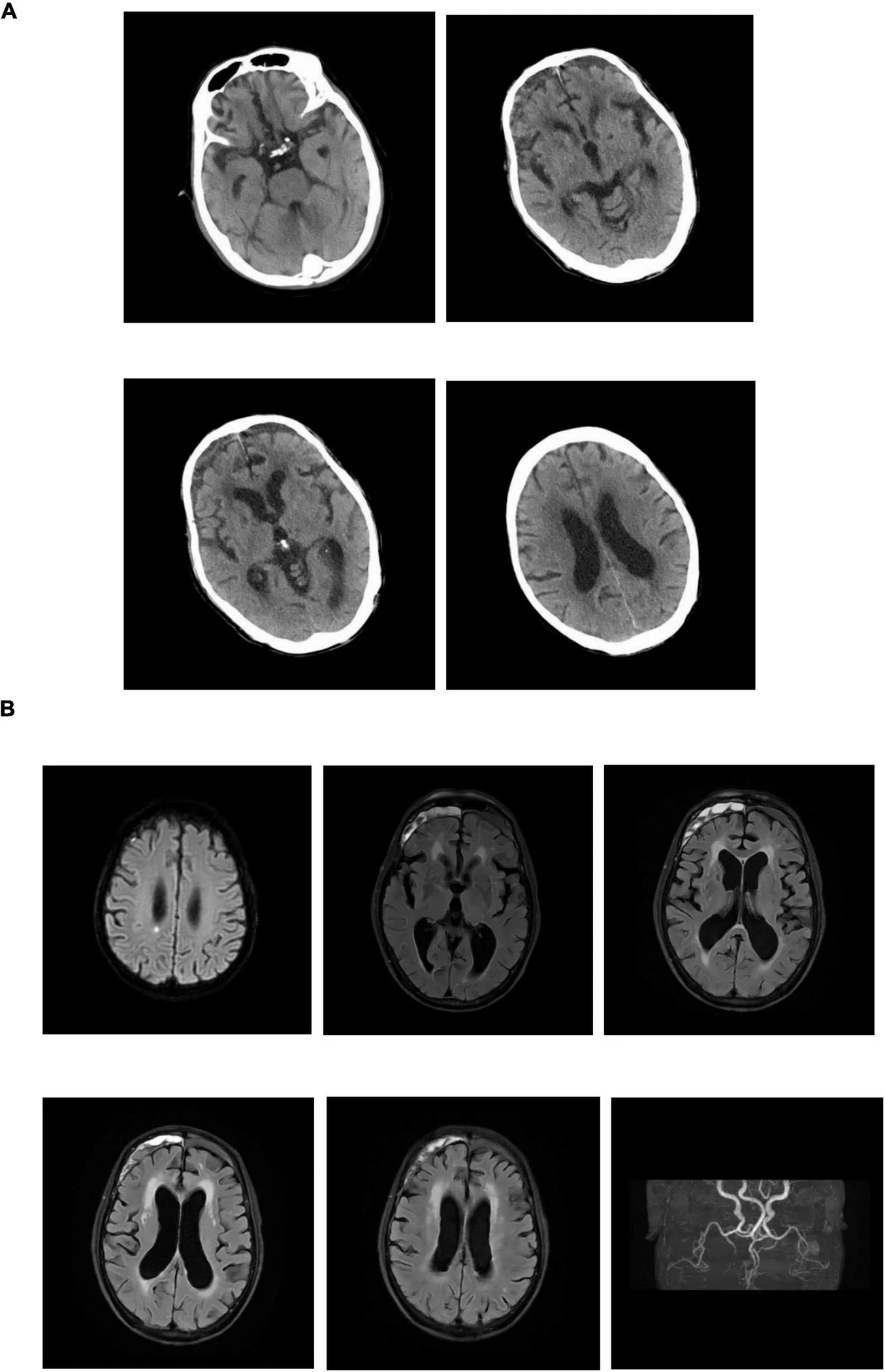
Neuroimaging findings after traumatic brain injury. **(A)** Non-contrast head computed tomography showed bilateral frontal subdural hematomas in the subacute-to-chronic stage, a small subarachnoid hemorrhage, a right occipital fracture, chronic lacunar infarcts in both basal ganglia, white matter demyelination, and age-related cerebral changes. **(B)** Brain magnetic resonance imaging with diffusion-weighted imaging and magnetic resonance angiography on a 3.0-T scanner showed a punctate diffusion-restricted lesion in the splenium of the corpus callosum, suspected contusion or acute lacunar infarction; encephalomalacia in the left temporal and right frontal lobes; multiple chronic lacunar infarcts involving the corpus callosum, bilateral basal ganglia, frontal and parietal lobes, centrum semiovale, and periventricular regions; ischemic demyelination; mild bilateral frontal subdural blood/effusion; and cerebral atherosclerosis.

On arrival, he was confused, had slurred speech, and gave irrelevant answers. Neurological examination was limited by poor cooperation. Pupils were equal and round, measuring 3.0 mm bilaterally, with brisk light reflexes. Neck stiffness was present. Spontaneous movements were observed in all limbs, but muscle tone and strength could not be reliably assessed; Babinski signs were negative bilaterally. Laboratory tests showed hyponatremia, with a serum sodium level of 125.6 mmol/L (reference range: 137–147 mmol/L), anemia, with a hemoglobin level of 91 g/L (reference range: 130–175 g/L), elevated carbohydrate antigen 19-9 (CA19-9) of 161.59 U/mL (reference range: 0.00–37.00 U/mL), and a positive fecal occult blood test (++). Non-contrast head computed tomography showed bilateral frontal subdural hematomas in the subacute-to-chronic stage, a small subarachnoid hemorrhage, a right occipital fracture, chronic lacunar infarcts in both basal ganglia, white matter demyelination, and age-related cerebral changes (Figure 1A). Brain magnetic resonance imaging with diffusion-weighted imaging and magnetic resonance angiography on a 3.0-T scanner showed a punctate diffusion-restricted lesion in the splenium of the corpus callosum, suspected contusion or acute lacunar infarction; encephalomalacia in the left temporal and right frontal lobes; multiple chronic lacunar infarcts; ischemic demyelination; mild bilateral frontal subdural blood/effusion; and cerebral atherosclerosis (Figure 1B). The initial diagnoses were post-traumatic brain syndrome and syndrome of inappropriate antidiuretic hormone secretion.

**FIGURE 2:**
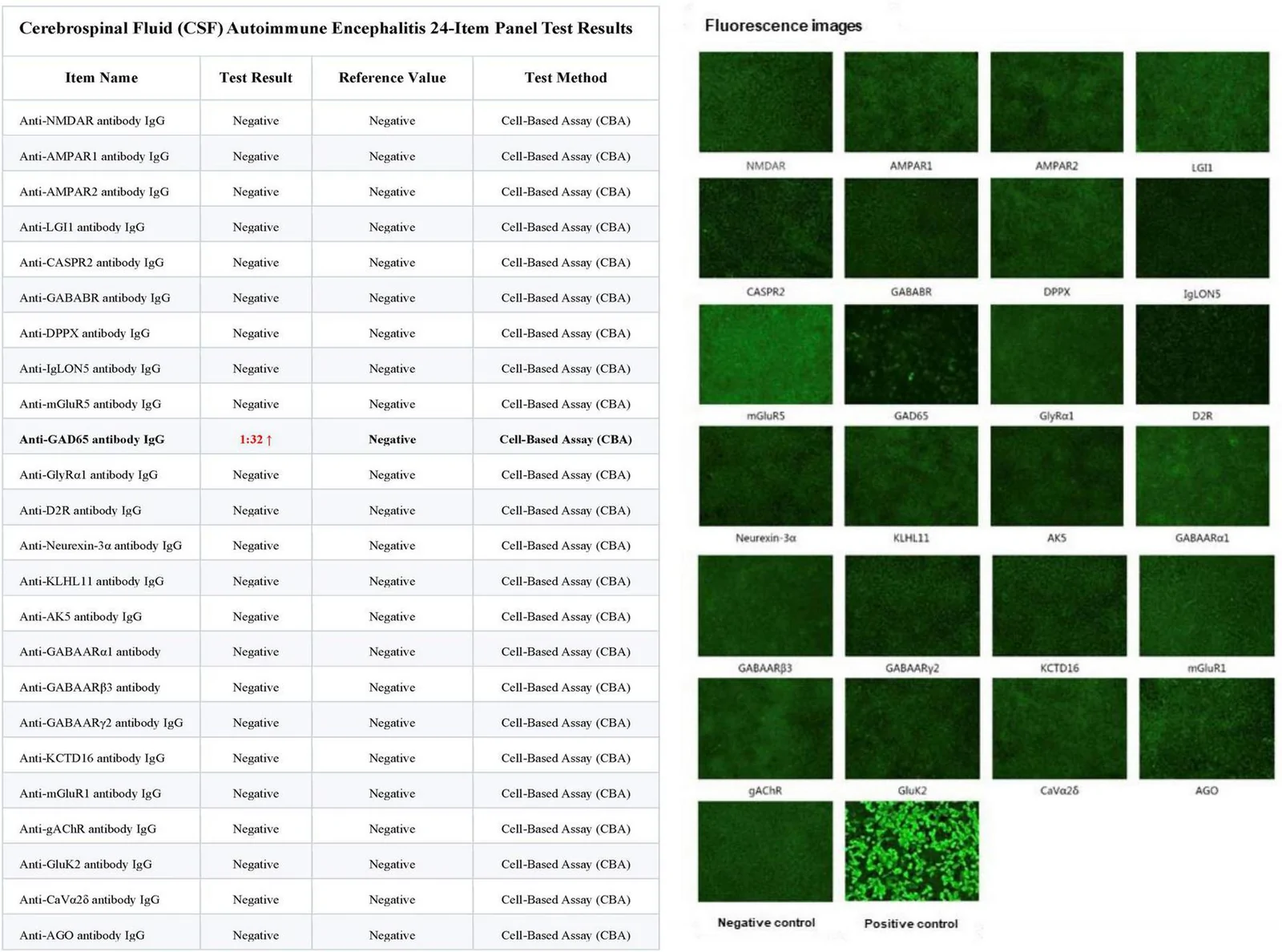
The CSF autoimmune encephalitis panel was positive for GAD65 antibody (titer 1:32).

**FIGURE 3:**
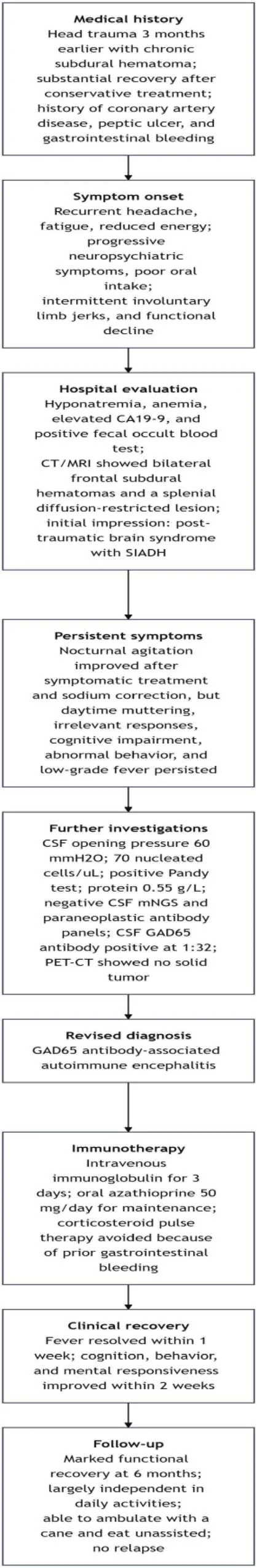
Schematic diagnostic pathway of the present case.

He received quetiapine 12.5 mg nightly, tolvaptan 7.5 mg once daily, memantine 10 mg once daily, atorvastatin 20 mg once daily, and nasogastric enteral nutrition. After 1 week, nocturnal agitation improved and serum sodium normalized, but daytime muttering, irrelevant responses, cognitive impairment, and intermittent low-grade fever persisted. Lumbar puncture showed an opening pressure of 60 mmH_2_O, a positive Pandy test, 70 nucleated cells/μL (reference range: 0–8 cells/μL), and cerebrospinal fluid protein of 0.55 g/L (reference range: 0.20–0.40 g/L). Cerebrospinal fluid paraneoplastic antibody testing, including 30 antibodies, and metagenomic next-generation sequencing were negative, whereas the autoimmune encephalitis panel in the cerebrospinal fluid (CSF) was positive for GAD65 antibody at a titer of 1:32 (Figure 2). The diagnosis was revised to GAD65 antibody-associated autoimmune encephalitis. Whole-body positron emission tomography-computed tomography showed no solid tumor. Because of his history of gastric ulcer and previous gastrointestinal bleeding, high-dose corticosteroid pulse therapy was avoided. Before initiating immunotherapy, the patient’s actual body weight was measured at 45.6 kg (the initial 48 kg recorded on admission was an estimate provided by the family due to the patient’s severe agitation). Based on this, he received intravenous immunoglobulin at a target total dose of 2 g/kg (90 g in total, administered as twelve 2.5 g/50 mL vials daily, totaling 30 g/day) for 3 consecutive days, followed by oral azathioprine 50 mg once daily as maintenance therapy. His temperature normalized within 1 week, and his mental status and responsiveness improved over the following 2 weeks. At 6-month follow-up, he was largely independent in daily activities, could ambulate with a cane, and could eat without assistance, with no relapse or serious treatment-related adverse events. The patient and his family reported marked improvement in communication, mobility, and daily functioning after immunotherapy, and the family noted that identifying a treatable autoimmune cause helped explain the prolonged deterioration after traumatic brain injury. The clinical course is summarized in Table 1, and the stepwise diagnostic reasoning is shown in Figure 3.

**TABLE 1:** Timeline of case events.

Date/time	Event/clinical course	Key findings/actions	Outcome
Approximately 3 months before admission	Head trauma	Fell while walking; chronic subdural hematoma was treated conservatively at a local hospital	Substantial neurological recovery
Approximately 10 days before admission	Symptom onset and progression	Recurrent headache, fatigue, reduced energy, agitation, reduced oral intake, intermittent involuntary limb jerks, and progressive cognitive and functional decline	Worsening mental and physical function
Day 0, admission	Hospital evaluation	Confusion; hyponatremia, anemia, elevated CA19-9, and positive fecal occult blood test. CT/MRI showed bilateral chronic subdural hematomas and a splenial diffusion-restricted lesion. The initial impression was post-traumatic brain syndrome with SIADH	Supportive treatment and symptomatic management initiated
Day 1–7	Persistent neuropsychiatric symptoms	Nocturnal agitation partially improved after symptomatic treatment and sodium correction, but daytime muttering, irrelevant responses, cognitive impairment, abnormal behavior, and low-grade fever persisted	Further evaluation was required
Day 8	CSF and etiological evaluation	CSF opening pressure was 60 mmH_2_O; nucleated cells 70/μL; Pandy test positive; protein 0.55 g/L. CSF mNGS and paraneoplastic antibody panels were negative. CSF GAD65 antibody was positive at a titer of 1:32. PET-CT showed no solid tumor	Autoimmune encephalitis was suspected
Day 9	Diagnosis revision	The diagnosis was revised to GAD65 antibody-associated autoimmune encephalitis	Immunotherapy was planned
Day 10–12	Immunotherapy	Intravenous immunoglobulin was administered for 3 days; oral azathioprine 50 mg/day was started for maintenance. Corticosteroid pulse therapy was avoided because of prior gastrointestinal bleeding	Fever resolved and cognition began to improve
Week 2	Recovery phase	Cognition, behavior, and mental responsiveness improved	Continued neurological improvement
Month 6	Follow-up	The patient showed marked functional recovery, was largely independent in daily activities, could ambulate with a cane, and could eat unassisted	Sustained recovery without relapse

## Discussion

Traumatic brain injury (TBI) may promote secondary neuroinflammation and disturb immune tolerance through mechanical disruption of the blood–brain barrier (BBB), allowing normally sequestered neuronal antigens to interact with the peripheral immune system and potentially initiate adaptive autoimmune responses (9, 10). This process involves microglial activation, complement signaling, and cytokine cascades, which may contribute to post-traumatic autoantibody formation and persistent cognitive or behavioral sequelae (6, 11). Although growing evidence supports an association between TBI and subsequent immune-mediated neurological disorders in susceptible individuals, a direct causal relationship with GAD65-associated autoimmune encephalitis (AE) remains unproven (12–14). Furthermore, determining the exact temporal causality in such cases can be clinically challenging. As is inherent to retrospective observations, an inverse correlation could theoretically be postulated: an insidious, slowly progressive onset of GAD65-associated AE might have induced subtle coordination or gait disturbances, which subsequently precipitated the fall and the TBI. However, in our patient, the family reported no preceding cognitive decline, psychiatric abnormalities, gait instability, or signs of stiff-person syndrome prior to the injury. The severe neuropsychiatric deterioration and inflammatory CSF changes emerged subacutely only after a clear latent period following the mechanical trauma. This clinical timeline strongly favors the hypothesis that the TBI served as an immunological trigger rather than a consequence of pre-existing AE, although a coincidental occurrence or a bidirectional relationship cannot be entirely ruled out.

In practice, the overlap between post-traumatic brain syndrome and AE may delay diagnosis. Clinical features that should raise suspicion for AE include subacute or fluctuating neuropsychiatric deterioration disproportionate to the structural lesion, persistent low-grade fever, mild cerebrospinal fluid (CSF) inflammation, and subtle or transient neuroimaging abnormalities (8, 14). In such situations, early CSF antibody testing, together with metagenomic next-generation sequencing (mNGS) to exclude infectious etiologies, may facilitate timely diagnosis (8, 15). GAD65-associated AE has a broad clinical spectrum extending beyond epilepsy and stiff-person syndrome to limbic and extralimbic cognitive-psychiatric presentations, and older patients may show a more insidious course (16, 17). Cerebrospinal fluid testing is generally more informative than serum testing, because intrathecal antibody synthesis or elevated CSF titers provide stronger support for pathogenic relevance than isolated low-titer serum positivity (18, 19). Brain magnetic resonance imaging (MRI) in GAD65-associated AE is often normal or nonspecific; when present, abnormalities may include subtle T2-weighted/fluid-attenuated inversion recovery hyperintensities in limbic regions or, less commonly, transient diffusion-restricted lesions in the splenium of the corpus callosum (20, 21). In the present case, the punctate diffusion-restricted splenial lesion was compatible with these uncommon but reported findings and could easily have been misinterpreted as post-traumatic or ischemic change (22).

This case has several practical implications. Recent TBI may obscure the onset of autoimmune encephalitis because headache, agitation, cognitive decline, and hyponatremia are easily attributed to post-traumatic or metabolic causes. However, persistent or fluctuating neuropsychiatric symptoms despite correction of these factors, particularly when accompanied by low-grade fever or imaging findings that do not fully account for the clinical picture, should prompt targeted CSF antibody testing (23). Mild CSF pleocytosis and modest protein elevation also require careful exclusion of infection. In this patient, negative metagenomic next-generation sequencing and paraneoplastic testing, together with phenotype-compatible CSF GAD65 antibody positivity, supported an autoimmune mechanism (18, 19). Compared with autoimmune encephalitis mediated by neuronal surface antibodies, GAD65-associated AE often shows a slower and less complete response to immunotherapy (17). Treatment should be individualized, especially in elderly patients or in those with contraindications to high-dose corticosteroids. Intravenous immunoglobulin (IVIG) can be considered for induction, followed when necessary by steroid-sparing maintenance therapy such as azathioprine, mycophenolate mofetil, or rituximab (24–26). Because this patient had a history of gastrointestinal bleeding, high-dose corticosteroid pulses were avoided; intravenous immunoglobulin induction followed by low-dose azathioprine maintenance was well tolerated and associated with marked functional recovery over 6 months. Although tumor association is uncommon in GAD65-associated AE, age-appropriate malignancy screening remains advisable. In this case, the isolated elevation of serum CA19-9 was considered a benign, non-specific finding related to his recent gastric ulcer and systemic metabolic stress. It is well documented that CA19-9 can be significantly elevated in older adults without malignancy, particularly in the setting of benign gastrointestinal inflammation or peptic ulcer disease (27). Continued follow-up is needed to monitor relapse, cognitive outcome, treatment tolerance, and occult malignancy.

## Conclusion

This case highlights the diagnostic challenge when autoimmune encephalitis mimics or overlaps with post-traumatic brain syndrome. While establishing definitive causality is difficult, unexplained subacute neuropsychiatric deterioration or persistent fever following a TBI should prompt clinicians to consider AE and pursue early CSF antibody testing. For elderly patients with GAD65-associated autoimmune encephalitis who are not suitable for corticosteroid therapy, a sequential regimen of intravenous immunoglobulin followed by azathioprine maintenance may be a safe and effective option.

## Data Availability

The original contributions presented in this study are included in this article/supplementary material, further inquiries can be directed to the corresponding author.
